# Supplementary material for: Time-resolved in situ visualization of the structural response of zeolites during catalysis
Source: Nat Commun. 2020 Nov 19;11:5901. doi: 10.1038/s41467-020-19728-3 (PMC7677390; doi:10.1038/s41467-020-19728-3)
Supplement: Supplementary file 1 — Supplementary Information [file 41467_2020_19728_MOESM1_ESM.pdf]

Supplementary Information: Time-resolved *in situ* visualization of the structural response of zeolites during catalysis

Jinback Kang[1], Jerome Carnis[1], Dongjin Kim[1], Myungwoo Chung[1], Jaeseung Kim[1], Kyuseok Yun[1], Gukil An[1], Wonsuk Cha[2,†], Ross Harder[3], Sanghoon Song[4], Marcin Sikorski[4], Aymeric Robert[4], Nguyen Huu Thanh[5], Heeju Lee[1,6], Yong Nam Choi[6], Xiaojing Huang[7], Yong S. Chu[7], Jesse N. Clark[8,9], Mee Kyung Song[5], Kyung Byung Yoon[5], Ian K. Robinson[10,11], Hyunjung Kim[1*]

[1]Department of Physics, Sogang University, Seoul 04107, Korea

[2]Materials Science Division, Argonne National Laboratory, Argonne, IL 60439, USA

[3]Advanced Photon Source, Argonne National Laboratory, Argonne, IL 60439, USA

[4]Linac Coherent Light Source, SLAC National Accelerator Laboratory, Menlo Park, CA 94025, USA

[5]Department of Chemistry, Sogang University, Seoul 04107, Korea

[6]Korea Atomic Energy Research Institute, Daejeon 34057, Korea

[7]National Synchrotron Light Source II (NSLS-II), Brookhaven National Laboratory, Upton NY 11973, USA

[8]Stanford PULSE Institute, SLAC National Accelerator Laboratory, Menlo Park, CA 94025, USA

[9]Center for Free-Electron Laser Science, Deutsches Elektronensynchrotron (DESY), 22607 Hamburg, Germany

[10]London Centre for Nanotechnology, University College London, WC1E 6BT, London, UK

[11]Condensed Matter Physics and Materials, Brookhaven National Laboratory, Upton NY 11973, USA

[†]Current Address: Advanced Photon Source, Argonne National Laboratory, Argonne, IL 60439, USA.

[*]e-mail: hkim@sogang.ac.kr

Supplementary Note 1. Coherent X-ray Diffractive Imaging

The coherent X-ray diffraction patterns collected in the far-field are directly correlated with the Fourier transform of the electron density of the samples. Since the detector measures the intensity, the phase information of the Fourier transform is lost. It means that the reconstruction of the real space image of the sample is impossible by performing an inverse Fourier transform of the diffraction pattern. However, the phase can be recovered through iterative method[1,2], namely by a 'phase retrieval algorithm'[3]. In this case, the sample size should be smaller than the coherence length of the incident X-ray beam dimensions.

Assuming that the electron density of the sample in real space is $f(\mathbf{x})$, its Fourier transform $F(\mathbf{k})$ is given by

$$F(\mathbf{k}) = \int_{-\infty}^{\infty} f(\mathbf{x})\, e^{(2\pi i \mathbf{k}\cdot\mathbf{x})} d\mathbf{x} \qquad (1)$$

where $\mathbf{x}$ is a real-space position vector and $\mathbf{k}$ is the reciprocal space vector in Fourier space. The phase retrieval algorithm, the iterative calculation of Fourier and inverse Fourier transform, assumes that the diffraction pattern has the $|F(\mathbf{k})|$ form. The derived electron density and its Fourier transform for the mth iteration represented as

$$G_m(\mathbf{k}) = |G_m(\mathbf{k})|\exp[i\phi_m(\mathbf{k})] = \mathcal{F}[g_m(\mathbf{x})] \qquad (2)$$

$$G'_m(\mathbf{k}) = |F(\mathbf{k})|\exp[i\phi_m(\mathbf{k})] \qquad (3)$$

$$g'_m(\mathbf{x}) = |g'_m(\mathbf{x})|\exp[i\theta'_m(\mathbf{x})] = \mathcal{F}[G'_m(\mathbf{k})] \qquad (4)$$

$$g_{m+1}(\mathbf{x}) = |f(\mathbf{x})|\exp[i\theta'_{m+1}(\mathbf{x})] = |f(\mathbf{x})|\exp[i\theta'_m(\mathbf{x})] \qquad (5)$$

where g_m, θ_m, and G'_m, ϕ_m are the estimates of real space components f, η, and reciprocal space components F, φ, respectively. In the estimation of $G'_m(\mathbf{k})$, the gathered diffraction intensity $|F(\mathbf{k})|$ is applied as a constraint.

In the error-reduction (ER) and hybrid input-output (HIO) approach[4], $g_{m+1}(\mathbf{x})$ are given by the following:

$$g_{m+1}(\mathbf{x}) = \begin{cases} g'_m(\mathbf{x}), & \mathbf{x} \in S \\ 0, & \mathbf{x} \notin S \end{cases} \quad \text{(ER)} \qquad (6)$$

where S is the expected sample size. The ER algorithm is suitable to find local minimum within a few iterations, but the error value reaches a 'plateau' after a large number of iterations, which could be misunderstood as the convergence point and is referred to as 'stagnation'[4].

$$g_{m+1}(\mathbf{x}) = \begin{cases} g'_m(\mathbf{x}), & \mathbf{x} \in S \\ g_m(\mathbf{x}) - \beta g'_m(\mathbf{x}), & \mathbf{x} \notin S,\ 0 \leqq \beta \leqq 1, \end{cases} \quad \text{(HIO)} \qquad (7)$$

During HIO, the former yields $g_m(\mathbf{x})$ outside of the support constraint, S, subtracting a portion of $g'_m(\mathbf{x})$. This algorithm is appropriate to find the convergence point with a large number of iteration[4]. Through sufficient iterations of HIO and ER, we can estimate both the real space electron density $f(\mathbf{x})$ and the full complex Fourier transform $F(\mathbf{k})$. In addition, genetic algorithms[5,6] can be applied to shorten the iterations and optimize the initial values. The shrink-wrap method[7] can also be applied to reduce the iteration numbers by varying the support constraint S based on the iterated function $g_{m+1}(\mathbf{x})$.

In Bragg coherent X-ray diffractive imaging (BCDI), the phase part of real space density $f(\mathbf{x})$, η, extracted by the phase retrieval algorithm, is construed as the displacement of lattice inside the sample[8,9]. We show the connection between this phase part and the displacement sensitivity in the following.

We start by assuming a 'not-strained' nanocrystal density in real space, $\rho(\mathbf{r})$, represented as the product of a lattice of infinite size, $l(\mathbf{r})$, cut with a shape function $s(\mathbf{r})$ that defines the shape and size of the crystal, where $\mathbf{r}$ is a real-space position vector[10].

$$\rho(\mathbf{r}) = s(\mathbf{r})l(\mathbf{r}) \qquad (8)$$

The lattice function can be expressed by a Fourier series,

$$l(\mathbf{r}) = \sum_{\mathbf{Q}} C_{\mathbf{Q}} \exp(i\mathbf{Q} \cdot \mathbf{r}) \qquad (9)$$

where $\mathbf{Q}$ is a reciprocal space vector and $C_{\mathbf{Q}}$ is the complex coefficient. If the lattice contains some local displacement represented as the displacement field, $\mathbf{u}(\mathbf{r})$, we have $\mathbf{r} \rightarrow \mathbf{r} + \mathbf{u}(\mathbf{r})$ and the lattice function is

$$\begin{aligned} l(\mathbf{r} + \mathbf{u}(\mathbf{r})) &= \sum_{\mathbf{Q}} C'_{\mathbf{Q}} \exp\left(i\mathbf{Q} \cdot (\mathbf{r} + \mathbf{u}(\mathbf{r}))\right) \\ &= \sum_{\mathbf{Q}} C'_{\mathbf{Q}} \exp(i\mathbf{Q} \cdot \mathbf{u}(\mathbf{r})) \exp(i\mathbf{Q} \cdot \mathbf{r}) \\ &= \sum_{\mathbf{Q}} G_{\mathbf{Q}}(\mathbf{r}) \exp(i\mathbf{Q} \cdot \mathbf{r}) \qquad (10) \end{aligned}$$

where $G_{\mathbf{Q}}(\mathbf{r})$ is defined as the product of a complex coefficient and the displacement factor. The Fourier transform of the strained lattice becomes

$$
\begin{aligned}
\hat{L}(\mathbf{q}) &= \int l(\mathbf{r}+\mathbf{u}(\mathbf{r}))\exp(-i\mathbf{q}\cdot\mathbf{r})d\mathbf{r} \\
&= \int \sum_{\mathbf{Q}} G_{\mathbf{Q}}(\mathbf{r})\exp(i\mathbf{Q}\cdot\mathbf{r})\exp(-i\mathbf{q}\cdot\mathbf{r})d\mathbf{r} \\
&= \sum_{\mathbf{Q}} \int G_{\mathbf{Q}}(\mathbf{r})\exp(i\mathbf{Q}\cdot\mathbf{r})\exp(-i\mathbf{q}\cdot\mathbf{r})d\mathbf{r} \\
&= \sum_{\mathbf{Q}} \hat{G}_{\mathbf{Q}}(\mathbf{q}) \otimes \delta(\mathbf{q}-\mathbf{Q}), \qquad (11)
\end{aligned}
$$

which represents alternation of the original lattice due to a displacement field. If the deformation goes to zero, $\mathbf{u}(\mathbf{r}) = 0$, $G_{\mathbf{Q}}(\mathbf{r}) = \text{const.}$ and $\hat{L}(\mathbf{q})$ becomes the simple reciprocal lattice of $l(\mathbf{r})$ by a series of δ functions.

When the size of the sample is within the transverse coherence length of the X-ray beam, the diffracted wavefield $\hat{\psi}(\mathbf{q})$ can be represented as the Fourier component of the entire nanocrystal $\rho(\mathbf{r})$. If the nanocrystal contains some lattice distortion, $\mathbf{u}(\mathbf{r})$,

$$
\begin{aligned}
\hat{\psi}(\mathbf{q}) &= \int \rho(\mathbf{r})\exp(i\mathbf{q}\cdot\mathbf{r})d\mathbf{r} \\
&= \int s(\mathbf{r})l(\mathbf{r}+\mathbf{u}(\mathbf{r}))\exp(i\mathbf{q}\cdot\mathbf{r})d\mathbf{r} \\
&= \hat{S}(\mathbf{q}) \otimes \hat{L}(\mathbf{q}), \qquad (12)
\end{aligned}
$$

from the former result of $\hat{L}(\mathbf{q})$, we get

$$
\begin{aligned}
\hat{\psi}(\mathbf{q}) &= \hat{S}(\mathbf{q}) \otimes \sum_{\mathbf{Q}} \hat{G}_{\mathbf{Q}}(\mathbf{q}) \otimes \delta(\mathbf{q}-\mathbf{Q}) \\
&= \sum_{\mathbf{Q}} \hat{S}(\mathbf{q}) \otimes \hat{G}_{\mathbf{Q}}(\mathbf{q}) \otimes \delta(\mathbf{q}-\mathbf{Q}) \qquad (13)
\end{aligned}
$$

The intensity recorded by the detector is given by

$$
\begin{aligned}
I(\mathbf{q}) &= |\hat{\psi}(\mathbf{q})|^2 \\
&= \left|\sum_{\mathbf{Q}} \hat{\psi}_{\mathbf{Q}}(\mathbf{q})\right|^2 \\
&= \left|\sum_{\mathbf{Q}} \hat{S}(\mathbf{q}) \otimes \hat{G}_{\mathbf{Q}}(\mathbf{q}) \otimes \delta(\mathbf{q}-\mathbf{Q})\right|^2 \qquad (14)
\end{aligned}
$$

which represents the diffracted intensity consisting of several diffraction peaks localized around reciprocal lattice points $\mathbf{Q}$. The shape of diffraction peaks is altered

by the Fourier transform of the shape function, $\hat{S}(\mathbf{q})$, and the additional displacement function, $\hat{G}_{\mathbf{Q}}(\mathbf{q})$. Since the cross-terms of the diffraction pattern are negligible, we can write

$$I(\mathbf{q}) = \sum_{\mathbf{Q}} \left|\hat{S}(\mathbf{q}) \otimes \hat{G}_{\mathbf{Q}}(\mathbf{q}) \otimes \delta(\mathbf{q}-\mathbf{Q})\right|^2. \qquad (15)$$

In the case of our experiment, we are interested in the diffraction around a particular **Q**, and we can introduce the diffracted intensity near the reciprocal point

$$\begin{aligned} I_{\mathbf{Q}}(\mathbf{q}) &= \left|\hat{\psi}_{\mathbf{Q}}(\mathbf{q})\right|^2 \\ &= \left|\hat{S}(\mathbf{q}) \otimes \hat{G}_{\mathbf{Q}}(\mathbf{q}) \otimes \delta(\mathbf{q}-\mathbf{Q})\right|^2. \qquad (16) \end{aligned}$$

Through Bragg coherent X-ray diffraction imaging, we can get the real space representation of $\hat{\psi}_{\mathbf{Q}}(\mathbf{q})$ via phase retrieval algorithm,

$$\begin{aligned} \rho_{\mathbf{Q}}(\mathbf{r}) &= \int \hat{\psi}_{\mathbf{Q}}(\mathbf{q}) \exp(i\mathbf{q}\cdot\mathbf{r}) d\mathbf{q} \\ &= \int \hat{S}(\mathbf{q}) \otimes \hat{G}_{\mathbf{Q}}(\mathbf{q}) \otimes \delta(\mathbf{q}-\mathbf{Q}) \exp(i\mathbf{q}\cdot\mathbf{r}) d\mathbf{q} \\ &= s(\mathbf{r}) C'_{\mathbf{Q}} \exp\big(i\mathbf{Q}\cdot\mathbf{u}(\mathbf{r})\big) \exp(i\mathbf{Q}\cdot\mathbf{r}) \\ &= |\rho(\mathbf{r})| \exp(i\phi(\mathbf{r})) \qquad (17) \end{aligned}$$

where $\phi(\mathbf{r})$ is the phase component of the real space function $\rho_{\mathbf{Q}}(\mathbf{r})$, given by

$$\phi(\boldsymbol{r}) = \phi_{C'} + \mathbf{Q}\cdot\mathbf{u}(\boldsymbol{r}) + \mathbf{Q}\cdot\mathbf{r} \qquad (18)$$

$\phi_{C'}$ is a constant from $C'_{\mathbf{Q}}$, $\mathbf{Q}\cdot\mathbf{r}$ is a phase ramp from the particular **Q** selection and $\mathbf{Q}\cdot\mathbf{u}(\mathbf{r})$ is a projection of the displacement field to the reciprocal lattice point. The constant term and the ramp term can be removed easily; only the projected displacement term remains. Therefore, we can interpret the phase component of the retrieved real space function as a projection of the displacement field in the reciprocal lattice point direction, given in this case by the particular Bragg point **Q** chosen.

In this study, 2D CXD patterns were collected to get the *in situ* changes of the internal distortion of the zeolite. In 2D retrieved images from the 2D CXD patterns, the electron density is summed and the displacement field is averaged along the direction perpendicular to the observation plane[11].

Starting from the definition of 3D CXD, the scattering intensity distribution of a Bragg peak in the kinematical approximation can be written as

$$I(\mathbf{q}) = \left|\sum_{n} s_n(\mathbf{q}) \exp(i\mathbf{q}\cdot\mathbf{r}_n)\right|^2, \qquad (19)$$

where the $\mathbf{r}_n$ and $s_n(\mathbf{q})$ are the position vector and the structure factor of the *n*th cell of the crystal, respectively. With the displacement vector $\mathbf{u}(\mathbf{r})$ to the crystal lattice vector $\mathbf{r}_n$, $I(\mathbf{q})$ can be rewritten as

$$I(\mathbf{q}) = \left|\sum_{xyz} s(x,y,z,\mathbf{q})\exp(i\mathbf{q}\cdot\mathbf{u}(x,y,z))\exp\big(i2\pi(hx+ky+lz)\big)\right|^2 \qquad (20)$$

where (x,y,z) and (h,k,l) coordinate are with the real lattice $(\mathbf{a},\mathbf{b},\mathbf{c})$ and the reciprocal lattice (**A, B, C**), respectively. If we consider 2D intensity slice, where $k=0$, it becomes[11]

$$I(\mathbf{q}) = \left|\sum_{xz}\left(\sum_{y} s(x,y,z,\mathbf{q})\exp\big(i\mathbf{q}\cdot\mathbf{u}(x,y,z)\big)\right)\exp\big(i2\pi(hx+lz)\big)\right|^2$$

$$\approx \left|\sum_{xz} \tilde{\rho}(x,z)\exp\big(i2\pi(hx+lz)\big)\right|^2 \qquad (21)$$

If $\sum_y s(x,y,z,\mathbf{q})\exp\big(i\mathbf{q}\cdot\mathbf{u}(x,y,z)\big)$ term varies slowly with $\mathbf{q}$, then one can approximate $I(\mathbf{q})$ as the Fourier transform of a discrete 2D object, $\tilde{\rho}(x,z)$. If the deviation of $\mathbf{q}$ from the investigated reciprocal lattice vector $\mathbf{G}$ is sufficiently small, $\phi(\mathbf{r}) = (\mathbf{q}-\mathbf{G})\cdot\mathbf{u} \ll 2\pi$, then $\tilde{\rho}(x,z)$ can be written as

$$\tilde{\rho}(x,z) = \sum_{y} s(x,y,z,\mathbf{G})\exp\big(i\mathbf{G}\cdot\mathbf{u}(x,y,z)\big). \qquad (22)$$

In the present experiment, the Cu ions are introduced in the ZSM-5 microcrystals by an ion exchange process which does not substitute Cu ion for Al atoms. Therefore the Cu ion cannot directly affect the structure factor $s(x,y,z,\mathbf{G})$.

However, the displacement field $\mathbf{u}(x,y,z)$ changes during the catalytic process of Cu ion. Then we can write $\mathbf{u}(x,y,z)$ as $\boldsymbol{u}(x,y,z)+\Delta\mathbf{u}(x,y,z)$. Then $\tilde{\rho}(x,z)$ can be written as

$$\tilde{\rho}(x,z) = \sum_{y} s(x,y,z,\mathbf{G})\exp\Big(i\mathbf{G}\cdot\mathbf{u}(x,y,z)\cdot\big(1+\beta(x,y,z)\big)\Big) \qquad (23)$$

where β is defined by the deformation coefficient, which is the strain-rate coefficient multiplied by the measurement time *t*.

$$\beta(x,y,z) = \frac{\Delta\mathbf{u}(x,y,z)}{\mathbf{u}(x,y,z)} = \alpha(x,y,z,t)\cdot t \qquad (24)$$

For the theoretical values, the structure factor $s(x, y, z, \mathbf{G})$ and displacement field $\mathbf{u}(x, y, z) \cdot (1 + \beta(x, y, z))$ are calculated for each element $(\Delta x, \Delta y, \Delta z)$ at (x, y, z) and sum over the y-direction.

The electron density might be changed due to $\Delta\mathbf{u}$ (even if it is very small) and $\Delta\mathbf{u}$ is varied at different y. Therefore, we cannot take the $s(x, y, z, \mathbf{q})$ out of the summation Σ_y.

The model we used in this study is that $s(x, y, z, \mathbf{G})$ has the form of a step function-like in x-, y-, and z-direction. Referring to Supplementary Fig. 7 and Supplementary Tables 2 and 3, propene non-absorbed regions have a constant structure factor C_{vac} and the propene absorbed regions also have other constants $C_{\mathrm{prop},n}$ $(n = 1, 2, 3, \cdots)$ with different order (n) of the adsorption of propene to match $\alpha(x, y, z, t)$ and $\beta(x, y, z)$ for specific positions. Thus the actual integration process in y-direction is made in a simple form:

$$s(x, y, z, \mathbf{G}) = \begin{cases} C_{\mathrm{vac}} & \{x, y, z\} \in A_{\mathrm{vac}} \\ C_{\mathrm{prop},1} & \{x, y, z\} \in A_{\mathrm{prop},1} \\ C_{\mathrm{prop},2} & \{x, y, z\} \in A_{\mathrm{prop},2} \\ \vdots & \vdots \end{cases} \qquad (25)$$

where A_{vac} and $A_{\mathrm{prop},n}$ denote the regions (shown in Supplementary Fig. 7) of propene non-absorbed and propene absorbed, respectively. Then the $\tilde{\rho}(x, z)$ becomes

$$\begin{aligned} \tilde{\rho}(x, z) = & \sum_{y \in A_{\mathrm{vac}}} C_{\mathrm{vac}} \cdot \exp\Big(i\mathbf{G} \cdot \mathbf{u}(x, y, z) \cdot \big(1 + \beta(x, y, z)\big)\Big) \\ & + \sum_{y \in A_{\mathrm{prop},1}} C_{\mathrm{prop},1} \cdot \exp\Big(i\mathbf{G} \cdot \mathbf{u}(x, y, z) \cdot \big(1 + \beta(x, y, z)\big)\Big) \\ & + \sum_{y \in A_{\mathrm{prop},2}} C_{\mathrm{prop},2} \cdot \exp\Big(i\mathbf{G} \cdot \mathbf{u}(x, y, z) \cdot \big(1 + \beta(x, y, z)\big)\Big) + \cdots. \quad (26) \end{aligned}$$

Note that the integration direction should be the exit beam direction in the actual experiment. However, in the present experiment, the angle between the exit beam and the y-direction is only ~ 3.6°. Assuming that the thickness in the y-direction is 1.0 μm, it corresponds to a path length difference of only 0.00197 μm, i.e., ~ 0.2 %. Therefore, we calculated $\tilde{\rho}(x, z)$ by summation along y-direction shown in Supplementary Equation 26.

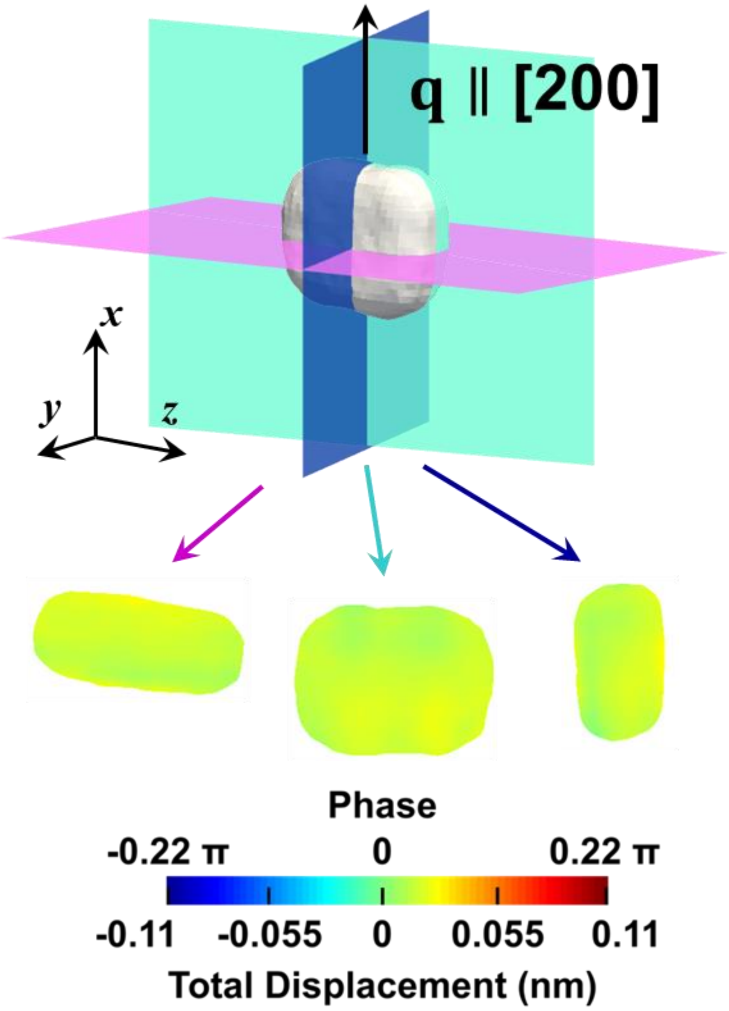

Supplementary Figure 1 | Three-dimensional image of the reconstruction of the Cu-ZSM-5 crystal. The 3D density is obtained by a phase retrieval process of the 3D CXD patterns at **q** || [200] taken at 250 °C in N_2 condition. Cross-sections in the *xy* (blue), *yz* (pink), and *zx* (cyan) planes are presented as 2D phase maps. The phases are directly proportional to the displacement in the crystal (set at 0 at the center of mass of the reconstructed 3D support). The maximum displacement in this condition does not exceed 0.025 nm, i.e., 2.5% of the (200) lattice constant.

Supplementary Note 2. Fourier Transform Infrared Spectroscopy measurement

Fourier-transform infrared spectroscopy (FTIR) was performed to confirm the processes shown in Fig. 1b. FTIR results (i.e., absorbance) are shown in Supplementary Fig. 2 at **a.** RT, **b.** 250 °C, and **c.** 400 °C in a vacuum, in propene, and in a mixture of NO and O_2. The detail peak assignments are shown in Supplementary Table 1.

Because the deoxygenation process does not occur at RT, the features of all reactants (NO, O_2, propene) are observed and the products (water and CO_2) are not observed 1 h after the insertion of NO and O_2 mixture. At 250 °C, the IR bands of N_2O_4, Cu-conjugated NO, propene, and the products are identified, directly indicating the deoxygenation process of NO_x with propene. They are observed immediately after the insertion of NO and O_2 mixture and vanished after 1 h. At 400 °C, the NO can be deoxygenated without hydrocarbons. Only IR bands of the products and propene are observed because the combustion process of propene by O_2 and direct deoxygenation process of NO occur independently of each other[12].

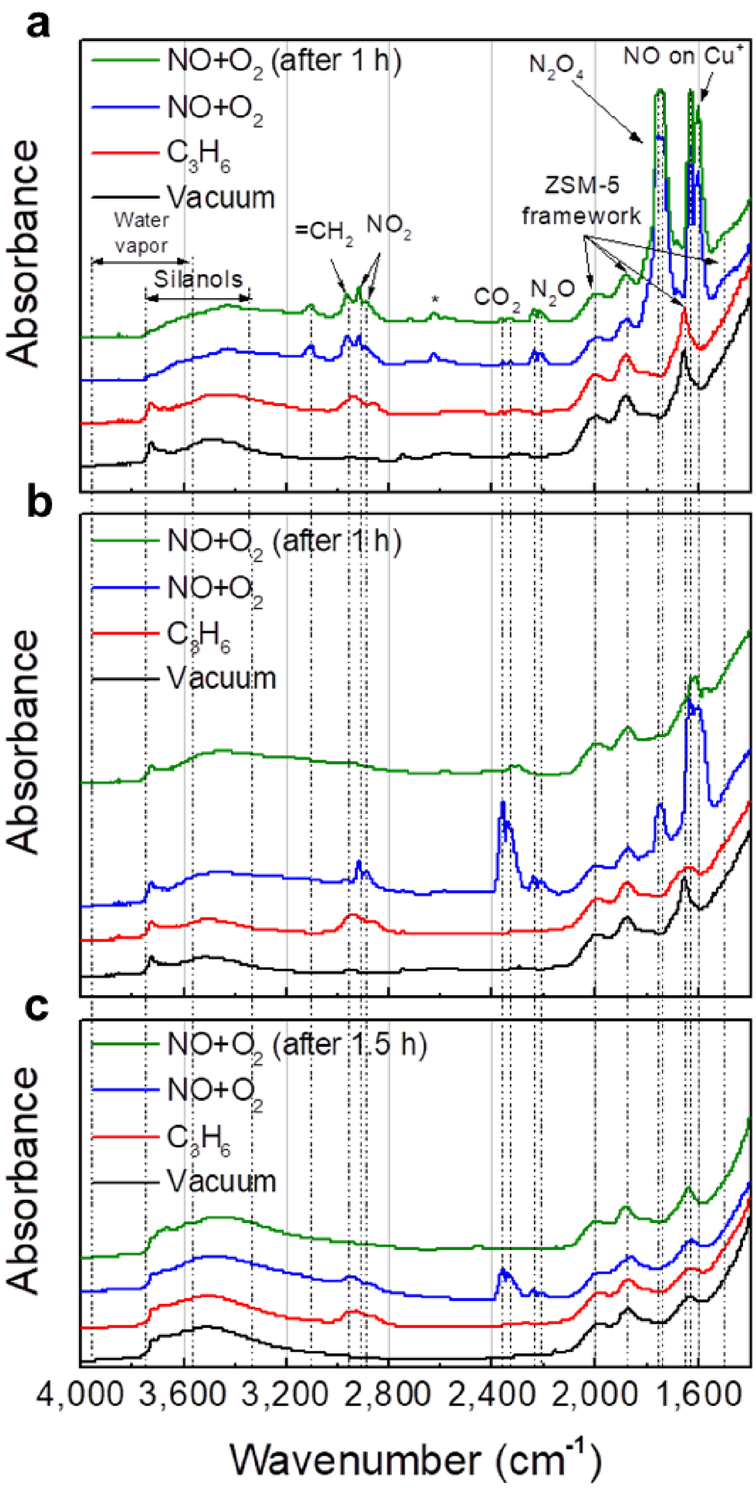

Supplementary Figure 2 | FTIR results from Cu-ZSM-5 during propene adsorption and NO$_x$ deoxygenation. The FTIR results from Cu-ZSM-5 powders at **a.** RT, **b.** 250 °C, and **c.** 400 °C. Individual peak assignments are presented in Supplementary Table 1. The powders were placed in a vacuum chamber. After

reaching the desired temperature, the spectrum was taken in a vacuum (black), and with propene (red). The excess propene molecules were removed by evacuation for 1 h and the spectra were then taken with NO and O_2 mixture (blue). After 1 ~ 1.5 h in NO and O_2 mixture condition (green), the final spectrum was measured to investigate if any changes due to the reaction could be observed. Before changing the temperature, the residual reactants were burnt at 400 °C under an excess O_2 environment for 30 min. The burnt residuals were removed by evacuation. Spectra are represented with a vertical offset for clarity.

Supplementary Table 1 | Peak assignments of FTIR results of Cu-ZSM-5 powder sample in Supplementary Fig. 2.

Band(cm^{-1})	Mode	Ref
1597, 1629	NO_2, NO in Cu^{2+}-(NO_2)(NO^-)	13, 14, 15
2000, 1890, 1630, 1460	ZSM-5 framework	15
1745, 1750	N_2O_4	13, 16
2214, 2236	N_2O	17
2325, 2355	CO_2	14, 18
2620	* Unknown	
2900, 2930	NO_2	19
2970	$\nu_{sym}(=CH_2)$	20, 21, 22
3108	$\nu_{asym}(=CH_2)$	20, 21, 22
3400 ~ 3700	ν(OH), zeolite channels	15, 22, 23
3500 ~ 3950	Water vapors	24

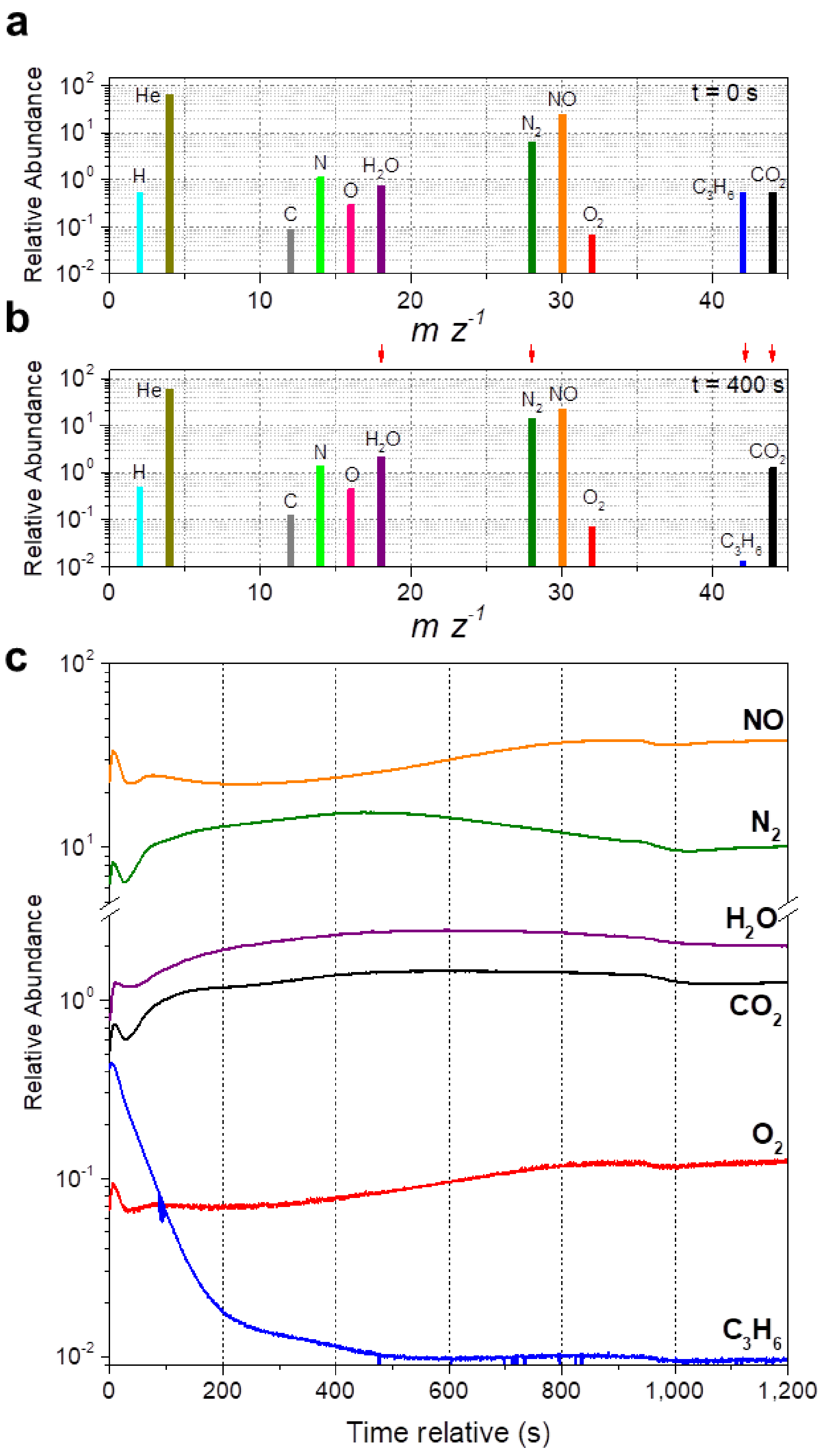

Supplementary Figure 3 | Product gas analysis by mass spectrometry The relative abundance of the reactants (NO (orange), O_2 (red), propene (blue)) and the products

(N_2 (green), CO_2 (black), and water (violet)) at **a.** t = 0 s and **b.** t = 400 s. At 400 s, significant changes in the abundance of N_2, H_2O, CO_2, and propene are observed. **c.** The relative abundance of the reactants (NO, O_2, propene) and products (N_2, H_2O, CO_2) as a function of time. They are monitored from the Cu-ZSM-5 powders in a quartz tube at 250 °C with a flow consisting of NO (5.00 cm^3 min^{-1}), O_2 (10.0 cm^3 min^{-1}), and He gas (35 cm^3 min^{-1}) to identify the products of the NO_x deoxygenation (process (ii)). The Cu-ZSM-5 powders were pre-exposed to propene (C_3H_6) flow (1.0 cm^3 min^{-1}) with He (49.0 cm^3 min^{-1}) for 1 h 40 min and flushed with pure He for 75 min. After the insertion of NO and O_2 in He, the relative abundance of propene decreases and those of water/nitrogen increase. The NO_x deoxygenation process was completed at ~1000 s.

Supplementary Note 3. Photon count threshold

We measured the CXD patterns for each single XFEL pulse. However, we determined the number of FEL pulses to average upon for defining optimized conditions between time resolution and the signal-to-noise ratio. From the CXD patterns in N_2, free from the reaction, we obtained reconstructions from the average of various numbers of pulses using the identical phase-retrieval algorithm procedures. The phase retrieval processes were performed in amplitude-free condition and shrink-wrap[7] for the support determination. The shape of the object, as determined by the reconstructed amplitude, remains persistent independently of the number of shots used so long as an average of 60 or more shots. Based on this result, we used a systematic averaging of 60 shots for each 2D CXD patterns, thus corresponding to a 0.5 s time resolution.

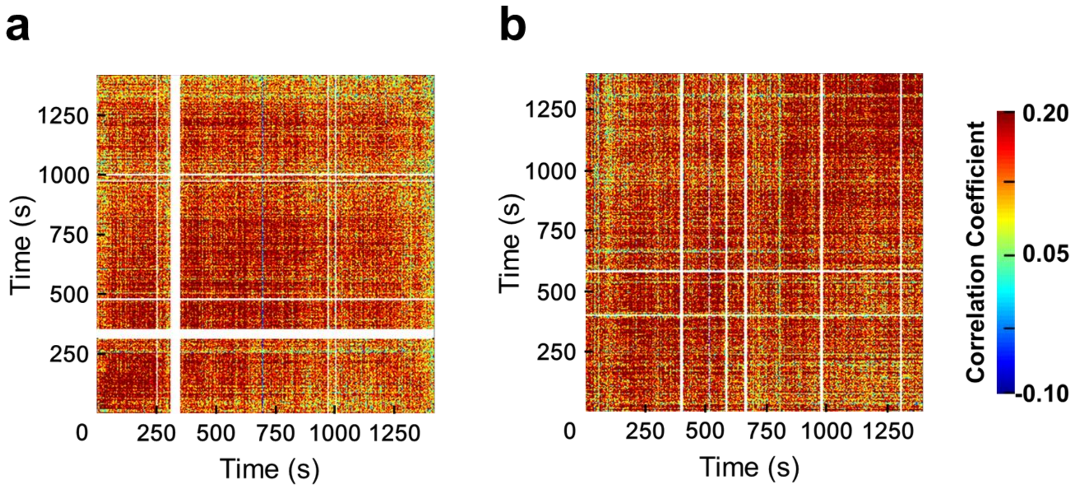

Supplementary Figure 4 | Cross-correlation of the reconstructed amplitude a. the propene adsorption (process (i)) and **b.** NO_x deoxygenation (process (ii)). The coefficients range from −0.1 to 0.2, where 1 indicates identical displacement, 0 no relation, and negative sign in the opposite direction. The white lines are due to missing data related to the inherent instability of the XFEL beam.

Supplementary Table 2 | Strain-rate coefficients (units: 10^{-6} s^{-1}) of Cu-ZSM-5 at 250 °C in different gas environments and time. The coefficients of $\alpha(t)_{(020)}$ are obtained from another Cu-ZSM-5 crystal in the CXD measurements described in the main text.

Environment	Time (s)	$\alpha(t)_{(200)}$	$\alpha(t)_{(020)}$
N_2	0 ~ 200	0.00247	0.00308
C_3H_6	0 ~ 762	-0.3637	-0.5046
	762.5 ~ 1400	-0.4878	-0.5046
$NO+O_2$	0 ~ 198.5	1.746	0.1282
	199 ~ 400	0.4880	0.1282
	400.5 ~ 1250	0.2196	0.1282
Vacuum	0 ~ 210	0.3364	0.1275

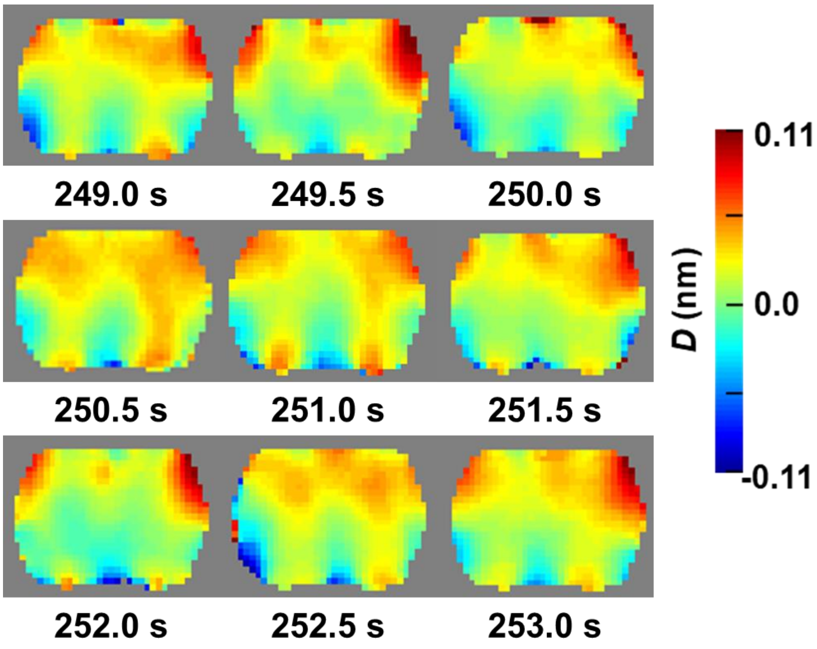

Supplementary Figure 5 | The displacement fields in the time range between 249 and 253 s with 0.5 s time resolution.

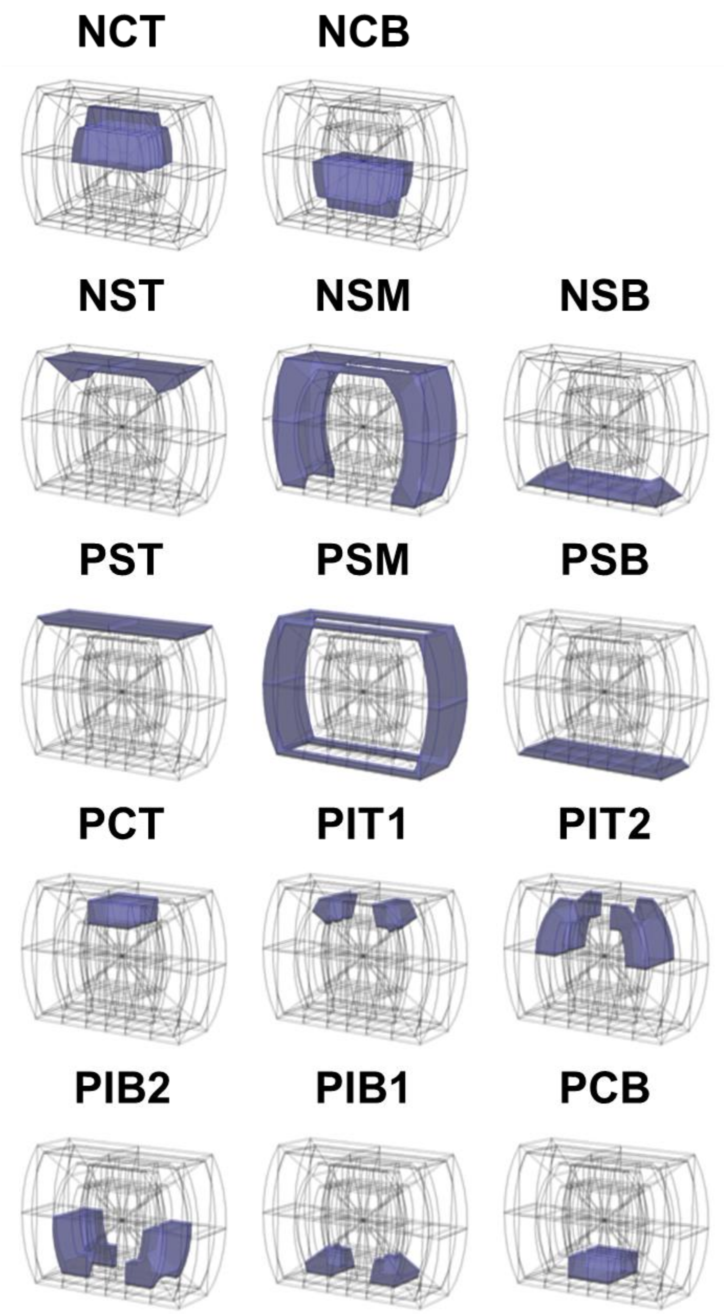

Supplementary Figure 6 | The model used in FEA simulation. Three characters represent the following. The first character denotes the section where the Cu sites are, i.e., where propene can be adsorbed: non-absorbed (N) or adsorbed (P). The second character denotes the part from the surface to inside: shell (S), intermediate (I), or core (C). The third character denotes the relative position along with the height: top (T), middle (M), or bottom position (B). The used strain-rate coefficients are shown in Supplementary Table 3.

Supplementary Table 3 | Strain-rate coefficients (units: 10^{-6} s^{-1}) of the labeled parts described in Supplementary Figure 6. The coefficients applied to the FEA simulation of the displacement at t = 250.5 s and 251.0 s. The proportional values of the $\alpha(t)_{(200)}$ and $\alpha(t)_{(020)}$ are applied for calculation at the state presenting describe the 'degree of adsorption of propene' at t = 0 ~ 762 s.

Part	t = 250.5 s		t = 251.0 s	
	$\alpha(t)_{(200)}$	$\alpha(t)_{(020)}$	$\alpha(t)_{(200)}$	$\alpha(t)_{(020)}$
**NCT**	0.1450	0.0546	0.1450	0.0546
**NCB**	0.1450	0.0546	0.1450	0.0546
**NST**	0.0650	0.0246	0.0650	0.0246
**NSM**	0.1250	0.0474	0.1250	0.0474
**NSB**	0.1250	0.0474	0.1250	0.0474
**PST**	-0.3368	-0.5046	-0.3368	-0.5046
**PSM**	-0.3368	-0.5046	-0.3368	-0.5046
**PSB**	-0.3368	-0.5046	-0.3368	-0.5046
**PCT**	-0.1440	-0.2157	-0.1460	-0.2187
**PIT1**	-0.1370	-0.2052	-0.1420	-0.2127
**PIT2**	-0.1370	-0.2052	-0.1420	-0.2127
**PIB2**	-0.1290	-0.1933	-0.1400	-0.2098
**PIB1**	-0.0990	-0.1483	-0.1400	-0.2098
**PCB**	-0.0840	-0.1258	-0.1250	-0.1872

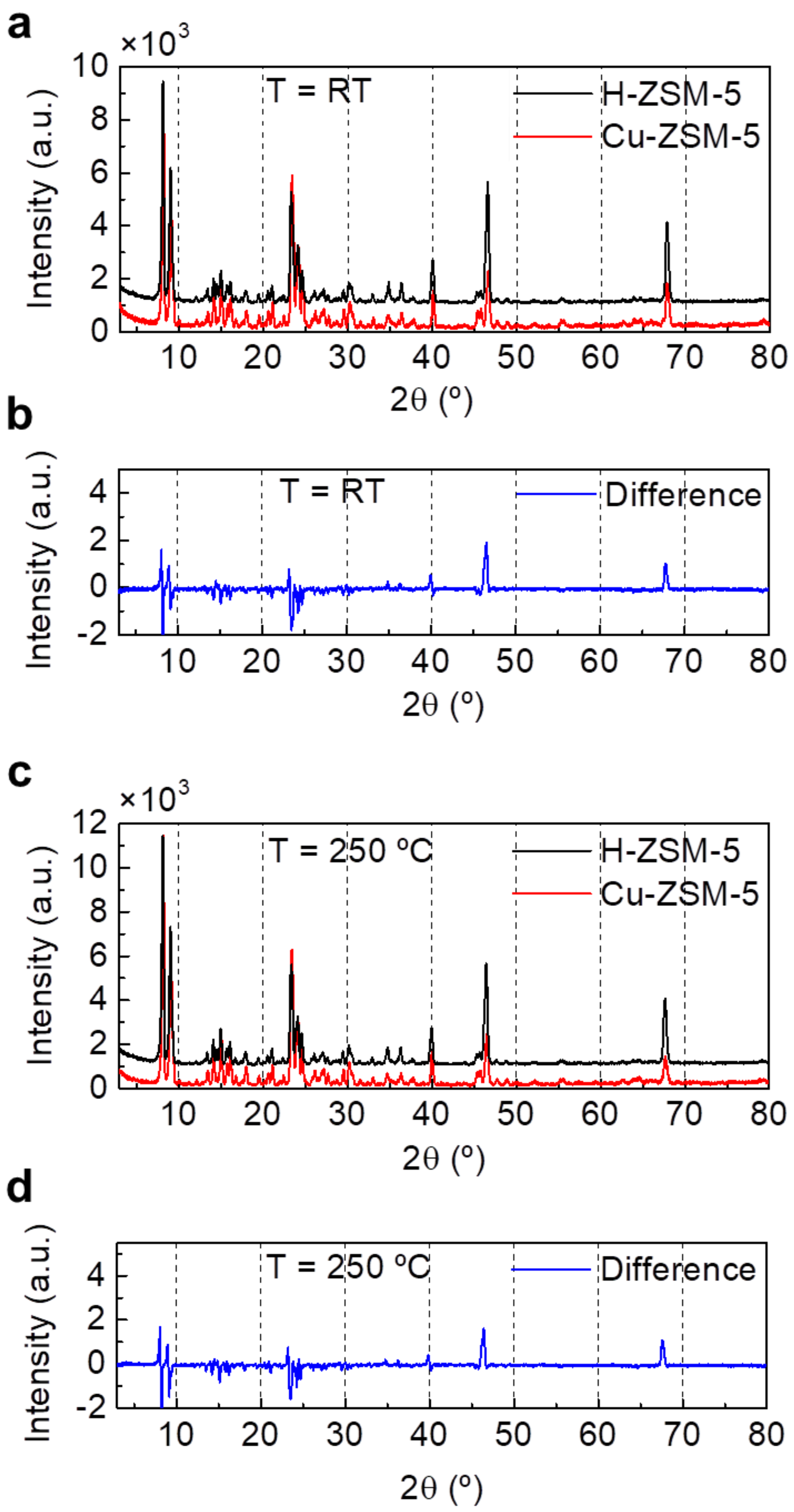

Supplementary Figure 7 | Powder diffraction of ZSM-5 samples. The beam energy is 8.048keV. **a.** The data of pristine ZSM-5 (H-ZSM-5) (in black), Cu-ZSM-5 (ion-

exchanged with $CuCl_2$ salt) (in red) and **b.** their difference (in blue) are shown at room temperature. At 250 °C, **c.** the data and **d.** their respective difference are shown as above. The data is shown with an offset for clarity. The lattice parameters of each sample are summarized in Supplementary Table 4. Both lattices have a monoclinic structure, $\alpha \neq 90°$ and $\beta = \gamma = 90°$.

Supplementary Table 4 | The lattice parameters from the powder diffraction

		Lattice Parameter					
		a (Å)	*b* (Å)	*c* (Å)	*α* (°)	*β* (°)	*γ* (°)
H-ZSM-5	RT	20.1210	19.9391	13.4221	90.6459	90.0000	90.0000
	250 °C	20.1234	19.9559	13.4255	90.6654	90.0000	90.0000
Cu-ZSM-5	RT	20.1209	19.9390	13.4188	90.3617	90.0000	90.0000
	250 °C	20.1238	19.9558	13.4254	90.3965	90.0000	90.0000

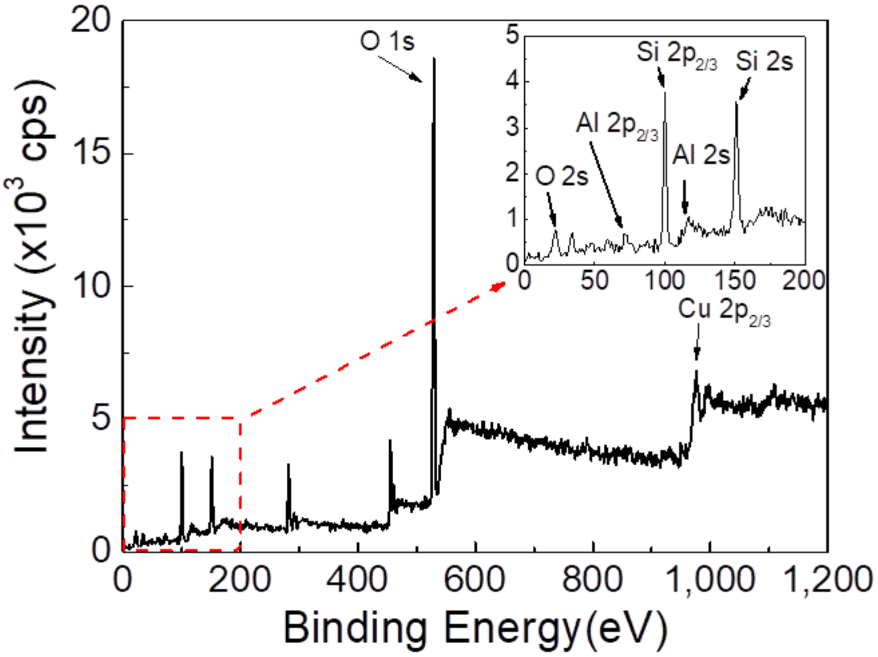

Supplementary Figure 8 | X-ray photoelectron spectroscopy of Cu-ZSM-5 with peak assignment for each element. For the Cu-ZSM-5 used in this study, the Si/Al molar ratio is estimated to be 14.99 and the Cu/Al is 0.6318. Chloride residuals were removed completely after the ion exchange with $CuCl_2$ since no peaks around the chloride position (198 ~ 202 eV) were observed. The inset shows the data expanded from 0 to 200 eV. The peaks with assignments show the orbital group of elements respective to the binding energy.

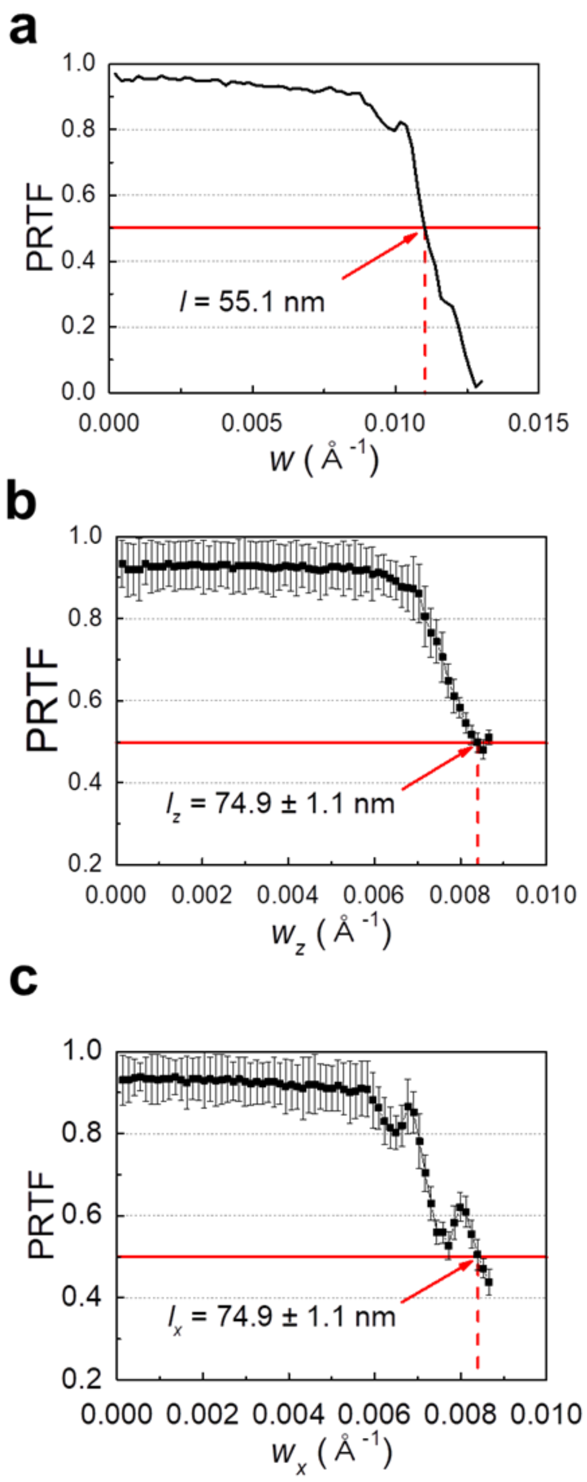

Supplementary Figure 9 | Phase retrieval transfer function (PRTF) plot of 3D and 2D reconstructions. a. The plot of PRTF for the 3D reconstruction. The spatial resolution (l) estimated is 55.1 nm. The PRTF plot of **b.** w_z and **c.** w_x in the 2D reconstruction. Both of the spatial resolutions of z- and x-direction, l_z and l_x, are 74.9 ± 1.1 nm.

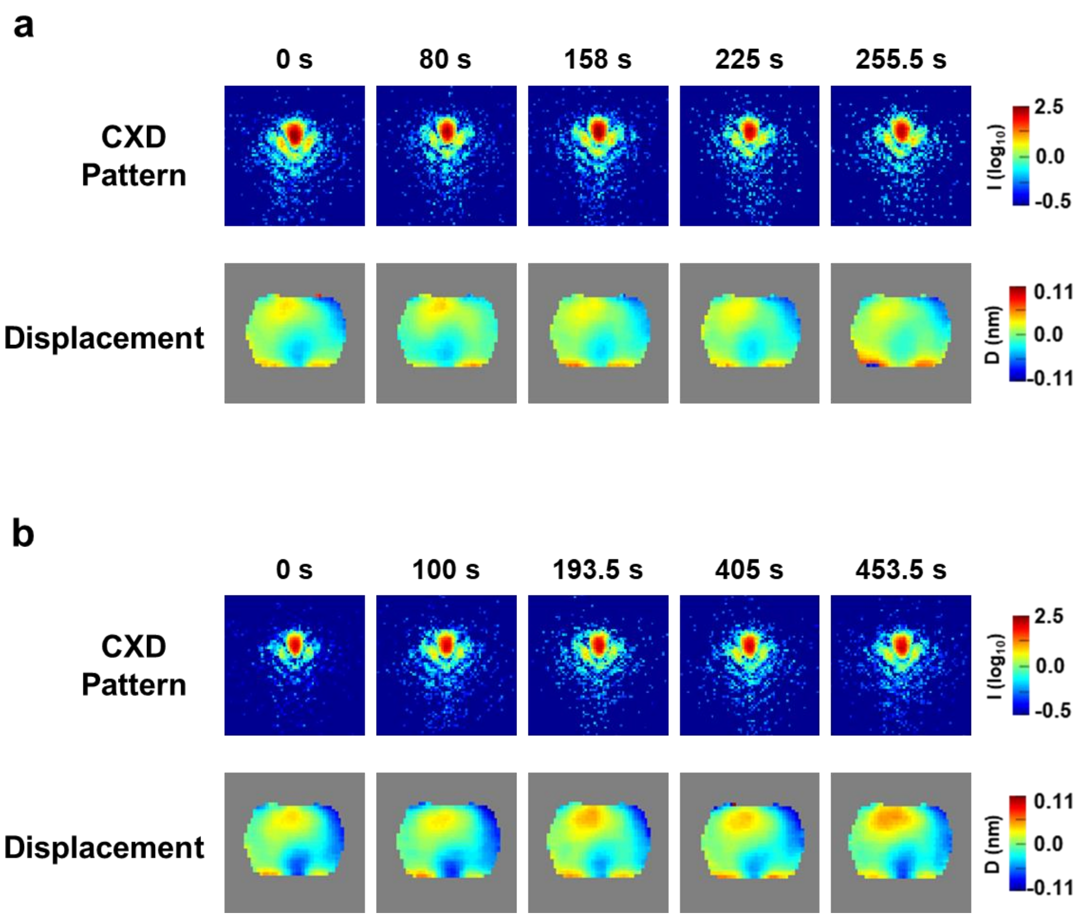

Supplementary Figure 10 | CXD patterns and reconstructed displacement maps of Cu-ZSM-5 at 250 °C a. in an N_2 environment. **b.** In a mixture of NO and O_2 without propene adsorption process.

Supplementary References

1. Miao, J., Charalambous, P., Kirz, J. & Sayre, D., Extending the methodology of X-ray crystallography to allow imaging of micrometre-sized non-crystalline specimens, *Nature* **400**, 342-344 (1999).

2. Williams, G. J., Pfeifer, M. A., Vartanyants, I. A., & Robinson, I. K., Three-dimensional imaging of microstructure in Au nanocrystals, *Phys. Rev. Lett.* **90**, 175501 (2003).

3. Fienup, J. R., Phase-retrieval algorithm for a complicated optical system, *Appl. Opt.* **32**, 1737-1746 (1993).

4. Fienup J. R., Phase retrieval algorithms: a comparison, *Appl. Opt.* **21**, 2158-2769 (1982).

5. Chen, C., Miao, J., Wang, C. W. & Lee, T. K., Application of optimization technique to noncrystalline X-ray diffraction microscopy: Guided hybrid input-output method, *Phys. Rev. B* **76**, 064113 (2007).

6. Colombo, A., Galli, D. E., De Caro, L., Scattarella, F. & Carlino, E., Facing the phase problem in coherent diffractive imaging via memetic algorithms, *Sci. Rep.* **7**, 42236 (2017).

7. Marchesini, S. et al. X-ray image reconstruction from a diffraction pattern alone, *Phys. Rev. B* **68**, 140101 (2003).

8. Robinson, I. K. & Harder, R., Coherent X-ray diffraction imaging of strain at the nanoscale, Nat. Mater. 8, 291-298 (2009).

9. Robinson, I. K. & Vartanyants, I. A., Use of coherent X-ray diffraction to map strain fields in nanocrystals, *Appl. Surf. Sci.* **182**, 186-191 (2001).

10. Clark, J. N. et al. Ultrafast three-dimensional imaging of lattice dynamics in individual gold nanocrystals, *Science* **341**, 56-59 (2013).

11. Labat, S., Chamard, V. & Thomas, O., Local strain in a 3D nano-crystal revealed by 2D coherent X-ray diffraction imaging, *Thin Solid Films* **515**, 5557-5562 (2007).

12. Hoost, T. E., Laframboise, K. A. & Otto, K., Co-adsorption of propene and nitrogen oxides on Cu-ZSM-5: an FTIR study, *Appl. Catal. B* **7**, 79-93 (1995).

13. Ayler, A. W., Larsen, S. C., Reimer, J. A. & Bell, A. T., An infrared study of NO decomposition over Cu-ZSM-5, *J. Catal.* **157**, 592-602 (1995).

14. Park, S., Park, Y., Park, S. & Kevan, L., Comparison of selective catalytic reduction of NO with C_3H_6 and C_3H_8 over Cu(II)-ZSM-5 and Co(II)-ZSM-5, *Phys. Chem. Chem. Phys.* **2**, 5500-5509 (2000).

15. Hwang, I. C., Kim, D. H. & Woo, S. I., The existence of dual Cu site involved in the selective catalytic reduction of NO with propene on Cu/ZSM-5, *Catal. Lett.* **42**, 177-184 (1996).

16. Zecchina, A. et al. Low-temperature Fourier-transform infrared investigation of the interaction of CO with nanosized ZSM5 and silicalite, *J. Chem. Soc. Faraday Trans.* **88**, 2959-2969 (1992).

17. Yuan, L., Toro, C., Bell, M. & Mullin, A. S., Spectroscopy of molecules in very high rotational states using an optical centrifuge, *Faraday Discuss.* **150**, 101-111 (2011).

18. Bell, V. A., Feeley, J. S., Deeba, M. & Farrauto, R. J., In situ high temperature FTIR studies of NO_x reduction with propylene over Cu/ZSM-5 catalysts, *Catal. Lett.* **29**, 15-26 (1994).

19. Pavlovich, M. J. et al. Air spark-like plasma source for antimicrobial NO_x generation, *J. Phys. D: Appl. Phys.* **47**, 505202 (2014).

20. Spoto, G. et al. IR study of ethane and propene oligomerization on H-ZSM-5: hydrogen-bonded precursor formation, initiation and propagation mechanisms and structure of the entrapped oligomers, *J. Chem. Soc., Faraday Trans.* **90**, 2827-2835 (1994).

21. Nakamoto, K., IR and Raman spectra of inorganic and coordination compounds, *Wiley*, 4th edn., New York (1986).

22. Jacobs, P. A., Framework hydroxyl groups of H-ZSM-5 zeolites, *J. Phys. Chem.* **86**, 3050-3052 (1982).

23. Vedrine, J. C., Auroux, A. & Coudurier, G., Combined physical techniques in the characterization of zeolite ZSM-5 and ZSM-11 acidity and basicity, *ACS Symp. Ser.* **248**, 253-273 (1984).

24. Wyatt, P. J., Stull, R. & Plass, G. N., The infrared transmittance of water vapor, *Appl. Opt.* **3**, 229-243 (1964).
